# Identified and potential internalization signals involved in trafficking and regulation of Na^+^/K^+^ ATPase activity

**DOI:** 10.1007/s11010-023-04831-y

**Published:** 2023-08-27

**Authors:** Rawad Hodeify, Sawsan Kreydiyyeh, Leen Mohammad Jamal Zaid

**Affiliations:** 1 Department of Biotechnology, School of Arts and Sciences, American University of Ras Al Khaimah, Ras Al Khaimah, United Arab Emirates; 2 Department of Biology, Faculty of Arts & Sciences, American University of Beirut, Beirut, Lebanon

**Keywords:** Na^+^, K^+^ ATPase, Trafficking, Endocytic motifs, Internalization, Phosphorylation, PGE2

## Abstract

The sodium–potassium pump (NKA) or Na^+^/K^+^ ATPase consumes around 30–40% of the total energy expenditure of the animal cell on the generation of the sodium and potassium electrochemical gradients that regulate various electrolyte and nutrient transport processes. The vital role of this protein entails proper spatial and temporal regulation of its activity through modulatory mechanisms involving its expression, localization, enzymatic activity, and protein–protein interactions. The residence of the NKA at the plasma membrane is compulsory for its action as an antiporter. Despite the huge body of literature reporting on its trafficking between the cell membrane and intracellular compartments, the mechanisms controlling the trafficking process are by far the least understood. Among the molecular determinants of the plasma membrane proteins trafficking are intrinsic sequence-based endocytic motifs. In this review, we (i) summarize previous reports linking the regulation of Na^+^/K^+^ ATPase trafficking and/or plasma membrane residence to its activity, with particular emphasis on the endocytic signals in the Na^+^/K^+^ ATPase alpha-subunit, (ii) map additional potential internalization signals within Na^+^/K^+^ ATPase catalytic alpha-subunit, based on canonical and noncanonical endocytic motifs reported in the literature, (iii) pinpoint known and potential phosphorylation sites associated with NKA trafficking, (iv) highlight our recent studies on Na^+^/K^+^ ATPase trafficking and PGE2-mediated Na^+^/K^+^ ATPase modulation in intestine, liver, and kidney cells.

## Introduction

The Na^+^/K^+^ ATPase (NKA) or sodium–potassium (Na^+^/K^+^) pump is ubiquitous in all animal cells but has different levels of expression [1]. It belongs to the family of P-type (phospho-intermediate type) ATPases, a family of proteins that have been evolutionarily conserved and present in both prokaryotes and eukaryotes [1–3]. Members of this family have a regulatory beta-subunit and a catalytic alpha-subunit with homologous catalytic sites. The alpha-subunit is membrane-bound and contains the binding sites for ATP, selected cation (s), and specific Na^+^/K^+^ ATPase inhibitors [1, 2].

The Na^+^/K^+^ ATPase consumes around 25% of the energy spent by the cell. It uses the energy derived from the hydrolysis of one ATP molecule to exchange two extracellular K^+^ ions for three cytoplasmic sodium ions [4]. The pump thus establishes and maintains a transmembrane sodium gradient used in the regulation of cell volume, pH, and in driving secondary active transport processes [5]. The gradient plays also a key role in the generation of action potentials in excitable tissues [6].

The central role of the Na^+^/K^+^ ATPase as an antiporter renders it essential for various cellular activities [7], and its presence in the membrane is mandatory for the accomplishment of the transport processes. The Na^+^/K^+^ ATPase is known to traffic between the cell membrane and intracellular stores, thus the regulation of its spatial and temporal distribution in the cell is crucial for the maintenance of homeostasis. Although previous studies reported trafficking as a mechanism of NKA regulation in several cell types [8–13], the molecular determinants of this process are still unclear. The pump residence in the plasma membrane depends on the rate of endocytosis and exocytosis. Internalization of the Na^+^/K^+^ ATPase was shown to be induced by various stimuli including hormones [14, 15], cardiac glycosides [16, 17], and anticancer agents [18, 19], and was shown to occur under pathological conditions such as hypoxia [10, 20], hypercapnia [21], and sepsis [22].

The NKA translocation appears to involve post-translational modifications and is directed by intrinsic sequence-based signal motifs. In this review, we discuss current knowledge on NKA trafficking. We also present a new analysis of the pump endocytic regulation, through screening of potential endocytic motifs and phosphorylation motifs in its catalytic alpha- subunit.

## Structure of the Na^+^/K^+^ ATPase

**Fig. 1:**
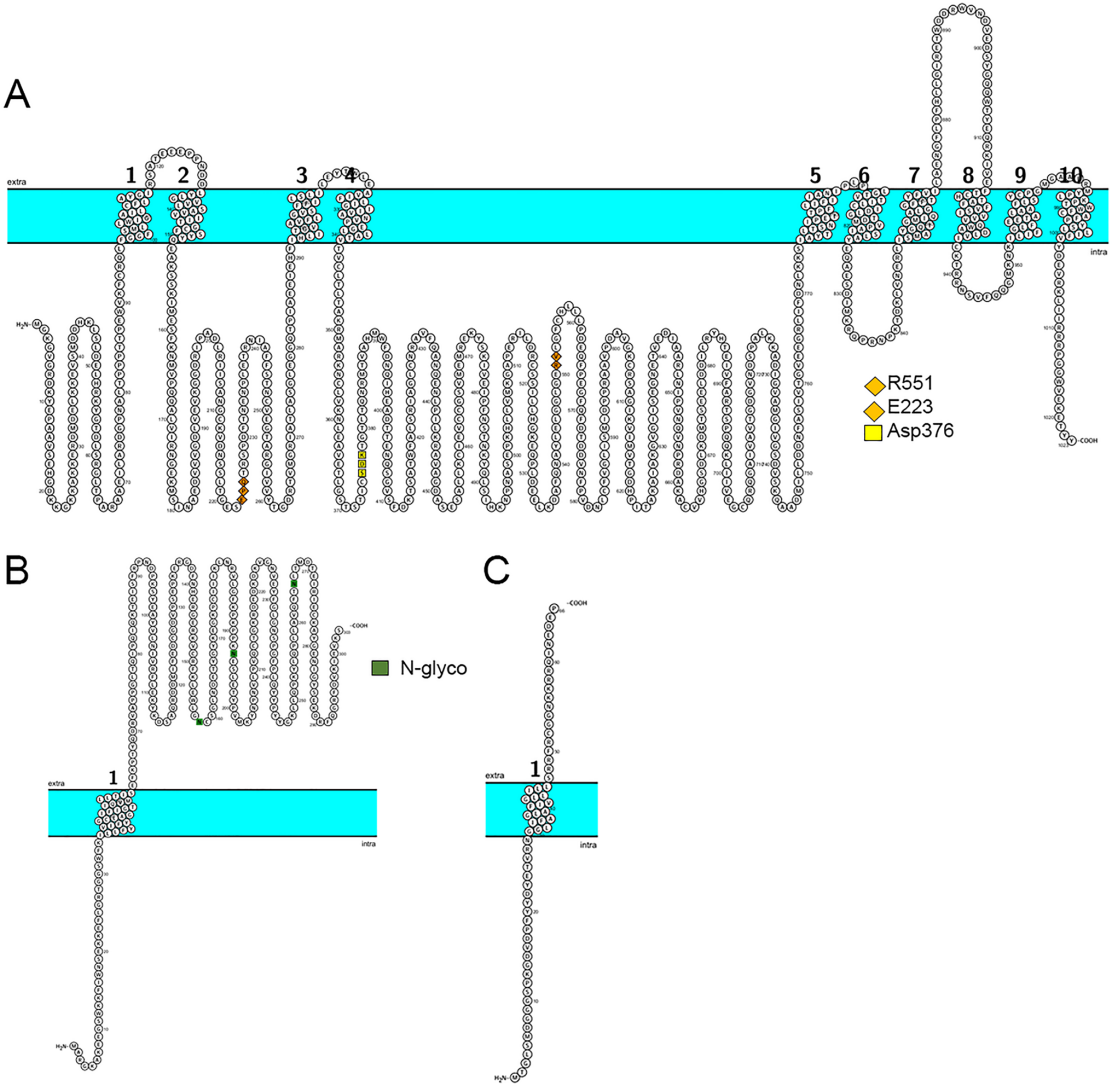
Topological representation of human sodium–potassium ATPase alpha, beta, and γ (FXYD)-subunits, using Protter software [24]. Topology model of human Na^+^/K^+^ ATPase alpha-subunit (**A**), beta-subunit (**B**), and γ-subunit (**C**). **A**: Human Na^+^/K^+^ ATPase alpha-subunit arranged in 10 transmembrane domains. The orange diamonds indicate glutamic acid (E223) and arginine (R551) forming the salt bridge which allows ATP binding. The yellow square indicates aspartate residue (D376) that is phosphorylated and dephosphorylated during each cycle. UniProt protein accession: P05023. Gene: ATP1A1. **B**: Topology of the human Na^+^/K^+^ ATPase beta-subunit, composed of 303 amino acids. The green squares indicate glycosylation sites, N158, N193, and N265. UniProt protein accession: P05026. Gene: ATP1B1. **C**: Topology of γ-subunit FXYD motif in the N-terminal extracellular end. UniProt protein accession: P54710. Gene: FXYD2

The Na^+^/K^+^ ATPase is a heteromeric protein composed of an alpha- and beta (β)-subunit in a 1:1 stoichiometry. A third gamma (γ)-subunit may be present [23] in some cells (Fig. 1).

The alpha (α)-subunit through its ATPase activity provides the energy needed for the uphill transport of sodium and potassium. The movement of ions from one side of the membrane to the other occurs through a conformational change of the enzyme, which cycles between two conformations: E1 and E2. In the E1 state, three sodium ions bind and the pump is phosphorylated. The resulting conformational change causes a change into the E2 state, in which sodium is released and two potassium ions attach. The phosphate group is then liberated and the pump reverts to the E1 state [7, 25].

The alpha-subunit, which performs the catalytic functions, has a molecular weight of around 110 kDa. It consists of approximately 1000 amino acid residues [26], arranged in 10 transmembrane domains (M1-M10), with 5 extracellular loops and 4 intracellular ones. Both the N- and C-termini reside in the cytosol [27]. This subunit has three cytoplasmic functional domains, the A domain (actuator), the N domain (nucleotide-binding), which contains the ATP binding site, and the P domain (phosphorylation domain) a substrate of the ATPase. The A domain acts as a phosphatase and consists of the segment (Gly^1^-Gln^88^) at the *N*-terminal end together with the loop between M2 and M3 [28]. It has been demonstrated that ATP binds at the salt bridge that spans between glutamic acid (E223) of the A domain and arginine (R^551^) on the N domain [29].

An aspartate residue (D^376^) in the P domain is phosphorylated and dephosphorylated during each cycle.

So far, four distinct isoforms of the alpha-subunits have been identified (alpha1, alpha2, alpha3, and alpha4). Although distinct subpopulations of alpha (alpha1 and alpha2) can exist within different regions of a single cell, the different isoforms generally show tissue or cell specificity [30].

Alpha1 is ubiquitously expressed and is the major housekeeping isoform that exists in most tissues. It is largely predominant in kidneys and most epithelia [26]. The isoforms alpha2 and alpha3 are found in neuronal tissue, skeletal muscle, and cardiac myocytes; alpha4 is expressed in the testis and modulates sperm motility [31].

Being a type II membrane protein, the beta-subunit of the NKA crosses the membrane only once and has a short cytosolic amino terminus and a large extracellular C -terminal end containing 6 cysteine residues forming three S–S bridges. Three sites (N^158^, N^193^, N^265^) of attachments for N-glycosylated oligosaccharide structures are also present in the extracellular domain [26, 32]. The human beta-subunit is composed of 303 amino acids with an overall molecular mass of ~35–55 kDa, depending on the glycosylation status [33]. Four isoforms have been detected so far: beta1, beta2, beta3, and beta(m). Beta1 is the most common isoform, beta2 was found in the nervous tissue while beta3 was identified in the testis and brain, and beta(m) in the heart and skeletal muscle [3]. Extracellular parts of the beta-subunit interact with the alpha-subunit at the level of the fourth extracellular loop. This interaction is crucial for accomplishing a normal ion transport, and alpha-subunit expressed alone showed almost no significant ATPase activity [34–36]. The beta-subunit plays an important role in the trafficking of the alpha-subunit, especially in its transport from the ER to the plasma membrane as well as in its correct insertion into the membrane [37, 38]. It helps also in maintaining the polarization of epithelial cells and prevents motility by dimerizing to a neighboring beta-subunit forming beta–beta bridges that enhance adhesion between cells [39]. Some evidence suggests that the beta-subunit plays also a role in ATP hydrolysis, as well as in ion transport and ouabain binding [40, 41].

In addition to the alpha- and beta-subunits, a gamma (γ)-subunit, a small-membrane protein of the FXYD family, associates specifically with the NKA in a tissue-specific manner [42, 43].

The FXYD protein families contains 7 members (FXYD1-7) that share all an FXYD motif (F: phenylalanine, X: any amino acid, Y: tyrosine, and D: aspartate) in the amino terminal end and two conserved glycine residues and one serine residue in the transmembrane domain. They are single span transmembrane domain proteins with the FXYD motif present on the extracellular amino terminal end [44]. The subunit is a 7–11 kDa type I protein that spans the membrane once and possesses an FXYD motif in the N-terminal extracellular end. Although it interacts with the NKA, it is not an essential component of the enzyme complex [43]. It does not play a role in the expression of the ATPase but can modify its transport properties by changing its affinity for its ion substrates and even for ATP, playing thus a fine-tuning role in the modulation of the pump’s activity [30, 44, 45].

The NKA subunits are synthesized independently in the ER where they are subsequently assembled [26]. Having different isoforms of each subunit allows for different combinations and variations in the pump between different tissues [39].

## Overview of trafficking pathways

The plasma membrane is a highly dynamic structure that undergoes continuous remodeling through multiple exocytosis and retrieval models [46]. These processes determine the spatiotemporal orchestration of membrane proteins and thus contribute to the regulation of many crucial biological functions associated with these proteins. In this section, we will present a brief summary of the exocytic and endocytic mechanisms and highlight the role of specific signals in these pathways.

### Exocytosis mechanisms

The exocytosis pathways are regulated by vesicular formation, transport through ER-Golgi, membrane targeting, docking, and vesicle fusion with the target membrane [47]. Coat proteins that are recruited to donor membranes are crucial players in this process. In the exocytotic pathway, coat protein complex I (COPI) and II(COPII), and clathrin, are the major coat proteins that facilitate the formation of most of the intracellular trafficking vesicles [48]. COPII is known to mediate anterograde transport from ER to Golgi through assembly of COPII complex containing five cytosolic proteins: the small GTPase Sar1p, the Sec23p/24p complex, and the Sec13p/Sec31p complex [49, 50]. Sec24p is crucial for coat assembly as well as for cargo selection, mainly binding proteins with specific signals such as the diphenylalanine motif [51]. After passing from ER to Golgi, cargo proteins are sorted at the trans-Golgi network (TGN) to be directed spatially to target membranes including apical and basolateral membranes and different endosomal compartments [52].

Clathrin-coated vesicles (CCV) mediate the transport of cargo molecules from the trans-Golgi network (TGN) to the membrane. Formation of CCV is mediated through the recruitment of several adaptor proteins and cargos proteins containing (D/E)XXXL(L/I) motif and LL motifs in the presence of Arf1-GTP and phosphatidylinositol-4-monophosphate (PI4P) [53, 54].

### Endocytosis pathways

For most integral membrane proteins, the availability at the plasma membrane is an output of regulation of the exocytosis process and retrieval process. Several endocytic pathways have been identified with clathrin-mediated endocytosis (CME) being the most understood. Accordingly, we will briefly summarize these processes of clathrin-dependent and -independent endocytosis. Clathrin-dependent endocytosis is initiated through the recruitment of AP-2 adapter proteins to plasma membrane-enriched phosphatidylinositol lipid, PI(4,5)P2, followed by binding of additional scaffolding proteins including FCH domain only (FCHO) proteins, EGFR pathway substrate 15 (EPS15), and intersectins [55]. The binding of AP-2 is mediated through endocytic motifs present in cytoplasmic tails of cargo proteins such as YXXΦ, LL motifs, or (D/E)XXXL(L/I) motif [48]. Another crucial step in the detachment of the clathrin-coated vesicles from the plasma membrane is mediated by the GTPase protein dynamin [56].

Caveolin-dependent endocytosis is another well-studied pathway in which caveolins are the main structural proteins in the formation of invaginations, known as caveolae, at the plasma membrane [57]. In this pathway, cholesterol-rich domain functions as a scaffolding domain for binding caveolins. In addition to caveolin complexes, several proteins were identified to be important in caveolae formation such as ATPase EHD2 [58]. Similar to CME, vesicular fission is dependent on dynamin. Like clathrin endocytic motifs, caveolin binding motifs have been reported. They are hydrophobic and rich in aromatic residues [59].

## Identification of potential trafficking motifs in the alpha-subunit of the Na^+^/K^+^ ATPase

Na^+^/K^+^ ATPase plasma membrane residence and activity are modulated by molecular mechanisms regulating exocytosis and endocytosis of the pump. Na^+^/K^+^ ATPase transition from ER to Golgi is in the form of Na^+^/K^+^ ATPase alpha and beta-assembled complexes packaged through COPII vesicles with assistance of spectrin and ankyrin complexes [60]. In the ER, both alpha- and beta-subunits undergo co-translation and post-translation maturation through interaction with ER chaperones [61–63]. During ER maturation, all isoforms of beta-subunit are *N*-glycosylated. The role of these glycosylations in the assembly of alpha and beta-complex and exit from ER is dependent on the type of isoform. While *N*-glycans were shown not to be involved in Na^+^/K^+^ ATPase alpha1: beta1 assembly, they were found to play an important role in the assembly of the alpha-subunit with the beta2-subunit [64]. Moreover, three disulfide bonds in the beta-subunit were demonstrated to differentially affect the assembly of alpha–beta complexes [33, 65]. Upon exit from Golgi, vesicles containing membrane proteins, including Na^+^/K^+^ ATPase, are targeted directly to plasma membrane through constitutive exocytosis, while other vesicles are targeted to plasma membrane only upon receiving a specific signal via regulated exocytosis.

Regulated exocytosis of Na^+^/K^+^ ATPase to the basolateral membrane has been shown to be initiated by dopamine in alveolar epithelial cells as well as upon activation of β-adrenergic receptors [66] and involvement of actin cytoskeleton [67, 68]. Hundal et al. (1992) demonstrated regulated translocation of isoform-specific Na^+^/K^+^ ATPase subunits from different intracellular compartments to the cell surface [69].

The same group followed up with an interesting study depicting insulin-dependent translocation in kidney cells using an exofacially epitope-tagged Na^+^/K^+^ ATPase alpha1-subunit [70].

On the other hand, several studies shed light on the different endocytotic processes that regulate the abundance of the pump at plasma membrane. In the Na^+^/K^+^ ATPase alpha-subunit, the caveolin binding motif (CBM) was identified in the proximity of the first transmembrane helix. Interestingly, the study by Wang et al. (2020) utilizing a point mutant of the CBM alpha1-subunit suggested that this CBM is not essential for the ATPase activity or plasma membrane residence [71]. Several other clathrin-independent pathways have been described [72] that depend on small GTPases such as dynamin, RhoA, Rac1, and Arf6. An additional endocytosis mechanism was dependent on flotillins, a group of proteins that are tightly associated with the inner leaflet of the plasma membrane [73]. Interestingly, studies demonstrated an association of flotillins with dynamin-dependent and -independent endocytosis [73, 74]. Reported also was the binding of flotillins with dileucine sorting signal in the trafficking of some proteins [75]. The association of different isoforms of the alpha-subunit with specific trafficking mechanisms is summarized in Table 1.

**Table 1:** Correlation between the tissue expression of different isoforms of the alpha-subunit with specific trafficking mechanisms

Na + /K + -ATPase alpha isoform	Cell line/tissue	Signal/trigger	Changes in the plasma membrane abundance (increased/reduced)	Key trafficking regulators	References
Alpha1	PTC cells OK cells	Dopamine	Reduced expression	PKC-dependent endocytosis	[76]
Alpha1	LLC-PK1 cells	Ouabain	Reduced expression	Clathrin-mediated endocytosis/ Src kinase-dependent	[77]
Alpha1	OK cells/A549 cells	Dopamine Hypoxia	Reduced expression	Tyrosine-based domain AP-2/Clathrin-dependent	[68, 78]
Alpha1	OK cells	AP-binding dileucine motif mutation/phorbol 12-myristate 13-acetate (PMA) treatment	Increased expression	Decreased AP-binding/clathrin-mediated shuttling PKC-dependent	[79]
Alpha1	A549 cells	Hypoxia/H_2_O_2_	Reduced expression	ROS-dependent endocytosis PKC-dependent	[20]
Alpha1	OK cells	Ouabain angiotensin II	Reduced expression	Decreased recruitment to the plasma via tyrosine-based adaptor protein 1sequence (IVVY-255)	[80]
Alpha1	OK	Coverslip-induced ischemia	Reduced expression	Role for [D/E]XXXL[L/I] Motif at position 494–499 in internalization	[81]
Alpha1 and alpha2	Skeletal muscle cells	Insulin	Increased surface expression	PI 3-kinase-dependent	[8]
Alpha1 and alpha2	Arterial vessels	Aldosterone induced a 40% decrease in both alpha1 and alpha2 isoform abundance in plasma membrane	Reduced expression	PKC-dependent phosphorylation microtubule-mediated trafficking	[82]
Alpha1 and alpha2	Ventricular myocytes	Short-term α-adrenoreceptor (α-AR) activation	Increased expression of alpha2	Actin-mediated trafficking Recycling from late endosomes to the plasma membrane	[83]
		Short-term β-adrenoreceptor (β-AR)	Reduced expression of alpha1	Increased α_1_- isoform expression in early endosomes	
Alpha1 and alpha2	Cell lines derived from LLC-PK1 cells	Membrane cholesterol reduction	Reduced expression	Cholesterol, Src, and Caveolin-1 dependent	[84]

Previous works revealed a connection between NKA activity and its plasma membrane abundance [76–84]. Chibalin et al. [76] using sucrose density gradient fractionation showed a decrease in the expression of the Na^+^/K^+^ ATPase on the surface of proximal convoluted tubular cells after dopamine treatment. The authors elegantly showed enhanced localization of NKA alpha-subunits in clathrin-coated vesicles, early, and late endosomes isolated from proximal convoluted tubular cells exposed to dopamine, as compared with untreated cells. The specificity of NKA recycling was supported by their data showing no change in the cellular distribution of glucose transporter type 4 and mannose 6-phosphate receptor, corroborating the assumption that changes in Na^+^/K^+^ ATPase are specific and not due to dopamine-induced bulk transport. Dopamine-mediated endocytosis was blocked in the presence of nocodazole, a microtubule depolymerizing drug, without disrupting the incorporation of Na^+^/K^+^ ATPase into clathrin-coated vesicles, suggesting a differential regulation of endocytosis and vesicular incorporation.

The specific recycling of plasma membrane proteins is known to be due to the presence of short-sequence motifs within their cytoplasmic regions allowing interaction with intracellular trafficking machinery [85]. Several endocytic motifs in clathrin-mediated internalization have been identified in a large number of plasma membrane proteins, including the canonical tyrosine-based motif, dileucine-based motif, NPxY (x is any amino acid), and several other noncanonical motifs [85, 86].

### AP-2

Several works focused on the identification of intrinsic domains within the Na+/K+ ATPase alpha-subunit allowing its binding to proteins of the endocytic machinery like adaptor protein-2 (AP-2). Through a series of site-directed mutagenesis using rat GFP-tagged Na+/K+ ATPase, Doné et al. [87] tested several potential endocytic motifs that were known in literature to bind AP-2 and revealed, through sequence analysis, the presence of several intracellular sites in the NKA alpha-subunit sequence for possible interaction with AP-2, including Y^50^KRH at the N-terminus, IVVY^255^ in the loop between TM 2 and TM3, and Y^469^IKT, Y^537^LEL, and ILRY^679^ in the loop between TM4 and TM5. The approach of the authors was based on the evidence for known binding motifs for two clathrin-associated protein complexes, specifically AP-2 -chain binding to a consensus NPXY or YppØ motif [81, 88], where Y designates tyrosine, X stands for any amino acid, Ø stands for hydrophobic amino acid, and p stands for positively charged residues. Testing the role of some identified potential endocytic motifs in dopamine-induced inhibition of the Na^+^/K^+^ ATPase in OK cells showed partial reversion of the inhibitory response only in cells with Na^+^/K^+^ATPase-Y^537^F mutant [78], suggesting that other additional mechanisms for pump internalization may be involved since the reversion was partial and not total. This partial reversion was not associated with changes in protein expression levels and probably occurred via a mechanism that does not affect the protein expression of the pump. The authors confirmed the essential role of this motif through a second mutation of tyrosine 737 to alanine, which completely blocked the inhibitory effect of dopamine on Na+/K+ ATPase activity. Interestingly, this study did not report the changes in the abundance of Na+/K+ ATPase molecules present in clathrin-coated vesicles isolated from the Y537A mutants despite the absence of immunoprecipitation with AP-2. The authors suggested that these resident NKA molecules in clathrin-coated vesicles originated from recycling endosomes in their shuttling to the plasma membrane. It also remains possible that an AP-2-independent recycling is involved in this process. In addition, Bonifacino and Traub [88] investigated the potential role of additional two dileucine-based motifs that were reported to bind to adaptor complexes of endocytic machinery including AP and other modulatory complexes [89]. This study did not present the evidence of changes in Na+/K+ ATPase at the plasma membrane in cells expressing L^499^A or L^554^A mutants but showed that these mutations did not alter the inhibitory action of dopamine or the stimulatory effect of phorbol esters, suggesting no major role for the two motifs in dopamine-mediated decrease in Na+/K+ ATPase activity.

### Arrestin and PP2A

Kimura et al. revealed the role of arrestin in the trafficking of the Na^+^/K^+^ATPase [90]. They showed through co-immunoprecipitation studies using mouse kidney tissues in situ binding of arrestins 2 and 3 and spinophilin to the Na^+^/K^+^ ATPase alpha-subunit and immunolocalization of the NKA and arrestin in intracellular compartments in COS cells. There was partial redistribution of NKA to the plasma membrane when arrestin 2 was co-expressed with spinophilin, suggesting an antagonistic role for spinophilin and arrestins in Na+/K+ ATPase internalization. An earlier study showed that spinophilin binds to TGN38 which is involved in recycling between the trans-Golgi network (TGN) and the plasma membrane [91]. Spinophilin is also known to bind to protein phosphatase 1 (PP-1), one of the phosphatases that regulate the activity of the Na^+^/K^+^ ATPase [92].

The direct binding of arrestin 2 to the large cytoplasmic loop of the Na+/K+ ATPase was shown through the pulldown experiments of GST fused arrestin 2 and GST- NKA-large cytoplasmic loop fusion [90]. Using a series of truncated mutants, the authors showed that the sequence of the first 53 amino acids of the large cytosolic loop between TM4 and TM5 is sufficient for binding to arrestin. However, constructs containing the first 155 and 175 aa of the M4-M5 cytosolic loop showed better bindings to arrestin 2, while the part containing 238 aa of the cytosolic loop showed lower binding capacity as compared to the 175, suggesting the presence within this segment of a binding site for spinophilin, another adaptor that was shown to inhibit arrestin 2 binding. Lecuona et al. [92] provided evidence for the direct interaction of PP2A with the first 90 aa of the N-terminus of the NKA ɑ-subunit. A few years later, Kimura et al. based on these findings pursued work on the regulation of Na^+^/K^+^ ATPase through arrestin’s binding. When truncated constructs of the NKA cytoplasmic loop 53 Δ, 155 Δ, and 175 Δ were used, PP2A binding was stronger than the binding to the full Na^+^/K^+^-ATPase cytoplasmic loop [90]. It was concluded that PP2A and arrestin compete for binding to the large cytoplasmic loop of the Na^+^/K^+^-ATPase.

### Clathrin and caveolin

Studies from Joseph I. Shapiro’s lab reported that ouabain induced, in LLC-PK1, Na^+^/K^+^ ATPase internalization through clathrin-dependent endocytosis through enhancing association between the Na^+^/K^+^ ATPase, adaptor protein-2 (AP-2), and clathrin [93]. In a following study using a similar model, the authors showed that ouabain-induced internalization is dependent on caveolin-1 [94]. By studying plasma membrane Na^+^/K^+^ ATPase through confocal imaging, and biochemically through biotinylation, Liu et al. showed that ouabain could not induce internalization of the pump [94] or the association of its alpha1-subunit with clathrin heavy chain and AP-2, when caveolin-1 was knocked down, suggesting an involvement of caveolin in the clathrin-dependent pathway [95–97]. However, whether caveolin is involved in the clathrin-independent endocytosis was not investigated. In the same study, Liu et al. [93] also reported reduced accumulation of Na^+^/K^+^ ATPase in early endosomes in the presence of methyl-beta-cyclodextrin, suggesting an important role for cholesterol in the ouabain-induced Na^+^/K^+^ ATPase internalization. A different study assessing NKA internalization in MDCK cells after energy depletion showed that the process is independent of caveolin-1 but dependent on AS160, a Rab GTPase-activating protein previously shown to play a role in the translocation of glucose transporter type 4 (GLUT4) [98]. Mapping the domain of interaction between AS 160 and Na^+^/K^+^ ATPase alpha-subunit, identified the cytoplasmic region between transmembrane domains 4 and 5 [99]. Morton et al. [100] reported that rat Na^+^/K^+^ ATPase alpha binds to COP-1 through a dibasic motif at position 54. This binding promotes the retrieval of the subunit in the ER. Mutating the dibasic motif enhanced the expression of mutant subunits at the plasma membrane, as shown by biotinylation studies and immunofluorescence [100].

### Identification of additional endocytic motifs

**Fig. 2:**
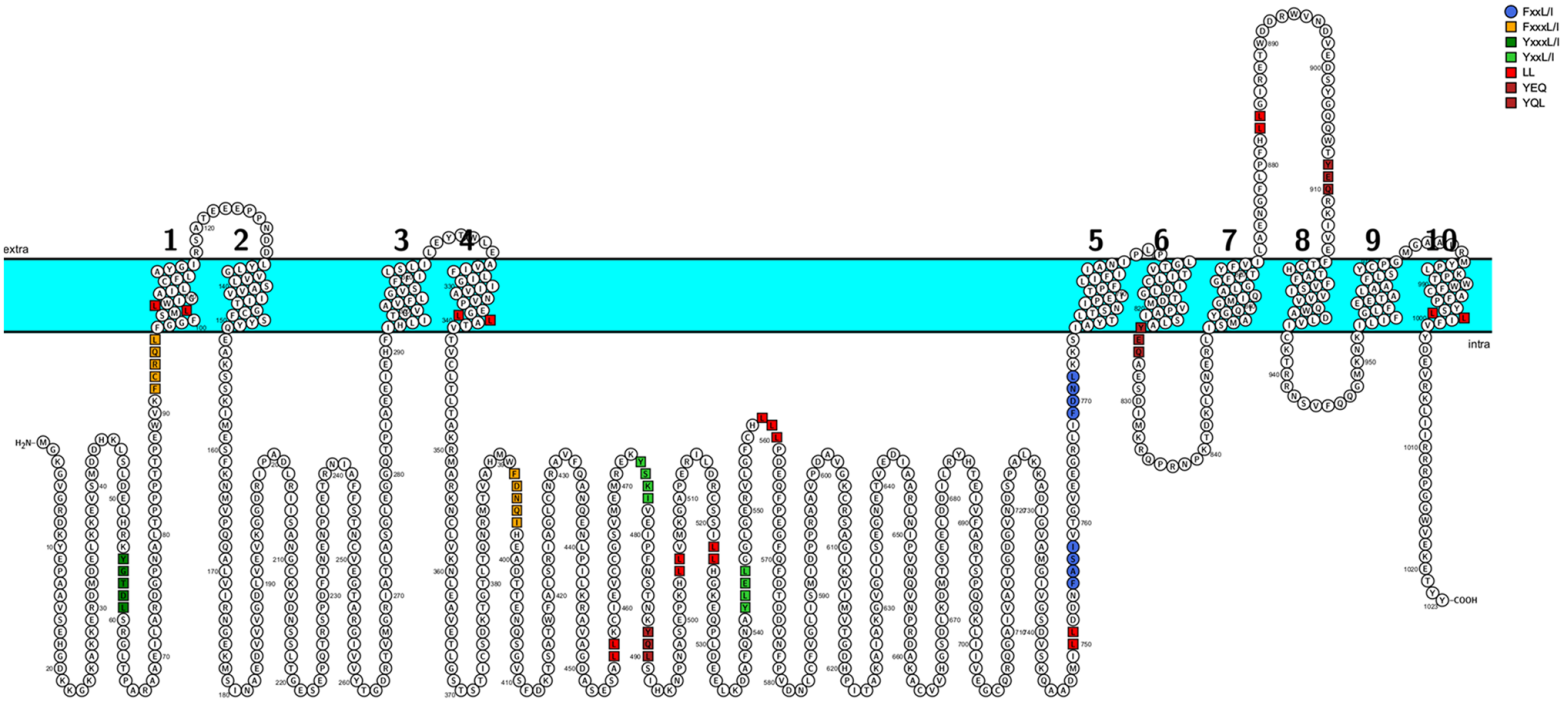
Known and predicted internalization signals in human sodium–potassium ATPase α-subunit. Motifs were identified based on canonical and noncanonical endocytic motifs reported in literature. The full sequence of the alpha-subunit was searched for tyrosine-based and dileucine-based motifs. The tyrosine-based motifs identified were either classical sequences including classical or non-classical tyrosine-based motifs. The green squares indicate classical tyrosine-based motifs including YxxL/I and YxxxL/I. Tyrosine-like based motifs such as FxxL/I and FxxxL/I are presented in blue circles (^769^FDNL and ^755^FASI) and orange squares (^92^FCRQL and ^393^FDNQI), respectively. The previously identified motifs at position 537 (^537^YLEL) is indicated in green squares. Non-classical tyrosine-based motifs are indicated by brick-colored squares (Y778TLTSN and Y824EQ). Dileucine-based motifs with similarity to [DE]xxxL[L/I] (ESALL^457^, EPQHLL^505^, DMILL^747^, EGRLI^768^) and L[L/I] (ENLPIL^438^, DIL^680^, SSILL^519^, ILL^749^, IL^897^) are indicted by red squares

In an attempt to identify the additional and still unrecognized potential internalization signals within Na^+^/K^+^ ATPase alpha, (Fig. 2), we searched human Na^+^/K^+^ ATPase alpha-subunit for canonical and noncanonical endocytic motifs based on the reported signals in the literature [78, 101, 102].

Within the N-terminus cytoplasmic domain, we identified the tyrosine-based YxxL motif [101] represented by (^55^YGTDL). Another YxxL-like sequence is also present at position ^92^FCRQL. Interestingly, we found no potential endocytic motifs in the cytoplasmic loop between transmembrane domains 2 and 3. In the large cytoplasmic loop between transmembrane 4 and 5, we identified several tyrosine-based and dileucine-based motifs. The potential tyrosine-based motifs are as follows: ^393^FDNQI, ^474^YAKI, ^488^YQLSI, ^542^YLEL, ^555^FCHL, ^683^YHTEI, ^755^FASI, and ^769^FDNL. Two other sequences, 778-YTLTSN and 824-YEQ, recognized by the presence of the critical tyrosine (Y) residue, could serve as a non-classical tyrosine-based sequence [102]. Similarly, several dileucine-based motifs with high similarity to consensus sequence [DE]xxxL[LI] were found in this cytoplasmic loop: ^453^ESALL^457^, ^500^EPQHLL^505^, ^747^DMILL, and ^764^EGRLI^768^. Other sequences with dileucine presence (L[L/I]) were also identified including ^438^ENLPIL, ^514^IL, ^519^SSILL, ^680^DIL, ^749^ILL, and ^897^IL.

Interestingly, studies by Pierre et al. group demonstrated increase in basal surface expression of Na^+^/K^+^ ATPase alpha1-subunits in cells expressing dileucine motif mutant L^499^V corresponding to the [D/E]XXXL[L/I] at position (500–505) identified in our screens [81].

**Fig. 3:**
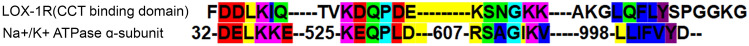
The homologous sequences of the CCT binding sites and human Na^+^/K^+^ ATPase alpha-subunit are shown. Similarities at three positions within N-terminus (32–37) and intracellular loops of Na + /K + ATPase alpha-subunit at positions 32–37, 525–530, and 607–612. A fourth similarity at position 998–1003 falls within a transmembrane domain

**Fig. 4:**
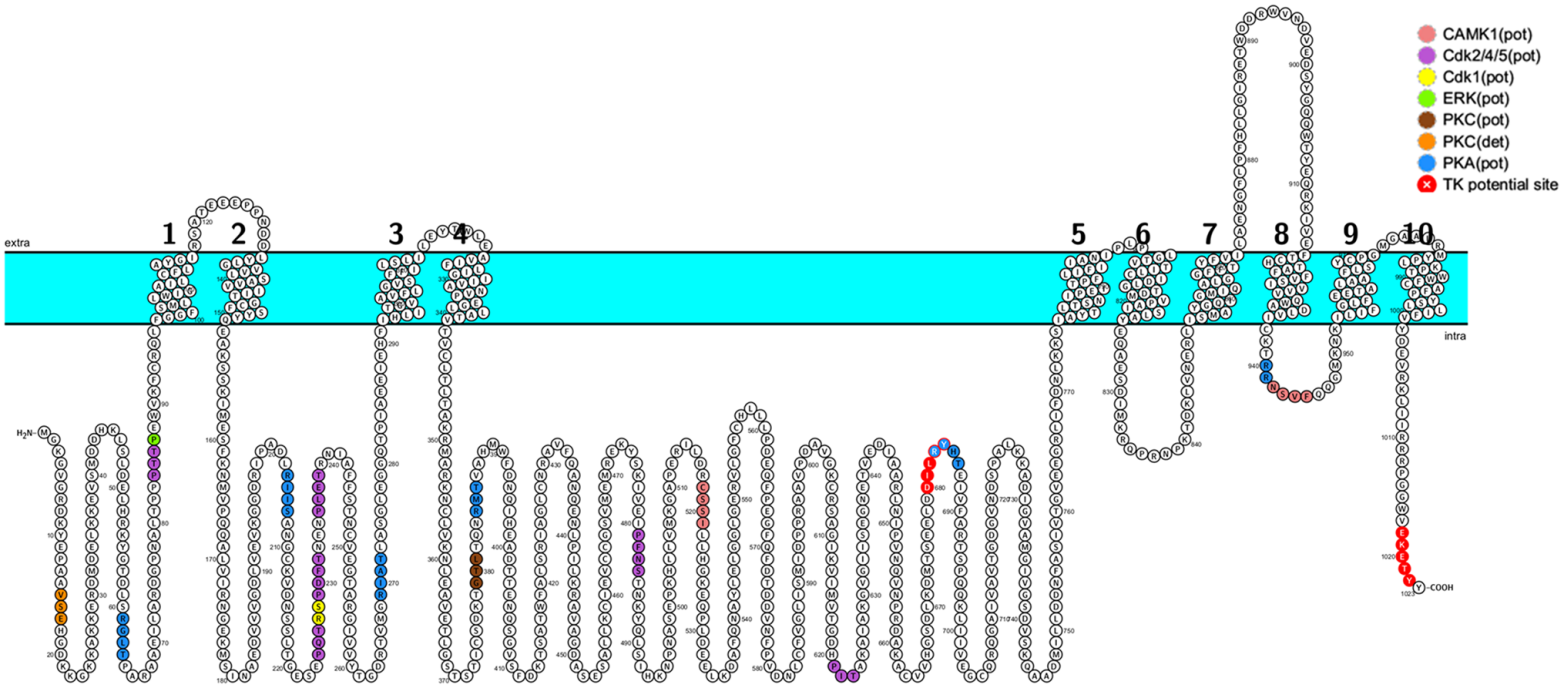
Reported and potential phosphorylation sites on human Na + /K + ATPase alpha-subunit. Minimal consensus motifs for serine/threonine kinases consist of having serine of threonine at position 0 and sequence similarity at positions − 1, − 2, and + 1(Rust and Thompson, 2011). Previously detected PKC phosphorylation site at serine18, PKC(det), is indicated in orange circles. Another PKC potential site, PKC(pot), is found at position and indicated with brown circles. Potential phosphorylation sites are shown—dark peach color for calcium/calmodulin dependent protein kinase I (CAMK1), violet for cyclin-dependent kinase 2/4/5 (cdk2/4/5), yellow for cyclin-dependent kinase 1 (cdk1), green for extracellular signal-regulated kinase (ERK), blue for protein kinase A (PKA), and red for tyrosine kinase (TK) site

Finally, driven by previous studies on the trafficking of human store-operated Ca2 + channel, Orai1 [103], we were curious to screen the NKA alpha-subunit for homologous sequences that can bind to chaperonin-containing TCP-1 (CCT). This chaperone is well known to be involved in the folding of several newly synthesized proteins [104] and was previously shown to regulate the trafficking of lectin-like oxidized low-density lipoprotein (LDL) receptor-1 (LOX-1 receptor) by binding to the cytoplasmic domain of the LOX-1 [105]. CCT was reported as a novel regulator of Orai1 internalization through binding to its intracellular loop, and inhibition of CCT-Orai1 interaction increases Orai1 residence in the plasma membrane [103]. Alignment of the LOX-1 receptor cytoplasmic domain sequence (MTFDDLKIQTVKDQPDEKSNGKKAKGLQFLYSPGGKG) with human NKA alpha-subunit shows significant homology at one position within the Na^+^/K^+^ ATPase alpha-subunit N-terminus (residues 32 to 37), and at two positions within cytoplasmic loop between transmembrane 3 and 4 (525-KEQPLDEE, 607-RSAGIKV) (Fig. 3). It remains possible that these three sequences serve as binding sites for CCT and consequently modulate the trafficking of the pump. A fourth similarity was detected at residues (998–1004, LLIFVYD. However, this sequence falls within a transmembrane domain and is unlikely to play a role in Na^+^/K^+^ ATPase alpha-subunit recycling (Fig. 4).

## Involvement of phosphorylation in the trafficking of the Na^+^/K^+^ ATPase alpha-subunit

Phosphorylation and dephosphorylation are the main processes regulating cellular activities including protein trafficking. Phosphorylation events affecting Na^+^/K^+^ ATPase structure and function have been extensively reported in the literature [106]. Phosphorylation may induce changes in NKA activity, binding of the pump’s subunits [107, 108], interactions with signaling proteins, and the recycling of the NKA alpha-subunit between the plasma membrane and intracellular compartments [33, 106]. However, the link between direct phosphorylation of NKA and its trafficking has been limited to few studies. In this section, we review the current knowledge derived from these works. We also propose distinct phosphorylation sites that may orchestrate NKA activity and/or trafficking, by presenting detected (det) and potential (pot) serine/threonine and tyrosine phosphorylation sites in NKA alpha-subunit.

### PKC phosphorylation sites and NKA internalization

Protein kinase C (PKC) is one of the earliest kinases known to negatively or positively modulate the Na^+^/K^+^ ATPase through a direct phosphorylation of its alpha-subunit [109]. Studies on rodent kidney cells have identified serine 11 and serine 18, located on the N-terminus of the alpha-subunit, as PKC phosphorylation sites [109]. Interestingly, while serine 11 is conserved in almost all mammalian kidney NKA alpha-subunits, serine 18 is missing in several mammalian species [110]. In proximal convoluted tubule cells, Chibalin et al. [14] demonstrated that the dopamine-induced internalization of the NKA alpha-subunit did not occur in the simultaneous presence of pharmacological inhibitors of PKC. Soon after this report, the same group used a truncated mutant of rat NKA alpha-subunit missing serine 11 and 18 and demonstrated that phosphorylation of Ser18 but not Ser-11 initiates internalization. Moreover, the mutant showed decreased abundance in clathrin-coated vesicles and early endosomes [78].

Another discovered protein kinase C phosphorylation site is serine 938 found within the cytoplasmic loop between the transmembrane regions M8 and M9 [111]. This residue is the same as serine 943 in full protein, and contrary to serine18, it is conserved in all mammalian Na^+^/K^+^ ATPase α-subunits. In OK cells exposed to the hormone angiotensin (ANG) II, Massey et al. [112] demonstrated that serine 938 phosphorylation stimulates the pump’s activity and increased its trafficking to the plasma membrane as revealed by the amount of biotinylated wild-type Na^+^/K^+^ ATPase alpha1 or S938A mutant in the presence or absence of ANG II [112].

### Adaptor proteins phosphorylation and NKA internalization

Other studies demonstrated an involvement of phosphorylation events in the dopamine-mediated NKA internalization through clathrin-dependent endocytosis. However, phosphorylation was linked to adaptor proteins implicated in the pump’s trafficking rather than NKA directly. On the other hand, AP-2 binding to tyrosine-based motif (Tyr-537LEL) located in the NKA alpha-subunit was shown to be vital for NKA endocytosis in response to dopamine or ROS [87]. In 2006, Bertorello’s laboratory reported that phosphorylation of adaptor protein AP-2 mu2 subunit mediates NKA internalization in OK and lung alveolar epithelial cells when treated with dopamine or ROS [113].

### Identification of potential phosphorylation sites inducing trafficking

In an attempt to identify the critical residues that can be phosphorylated and consequently modulate NKA activity and/or trafficking, we highlight the reported and potential phosphorylation sites within intracellular loops of the pump which could be of interest in future studies. We searched the sequence of alpha-subunit for serine/threonine protein kinase [114] and tyrosine kinase consensus sequences [115, 116] and highlighted the minimal consensus motifs for serine/threonine kinases presented as serine or threonine at position 0 and sequence similarity at position − 1, − 2, and + 1. For tyrosine kinases, we highlight the sites with [DE]-x(3)-Y. Future studies will identify which of those potential sites are bona fide phosphorylation sites and which can be directly involved in NKA trafficking.

## External signals and Na^+^/K.^+^ ATPase trafficking: the case of PGE2

Prostaglandin E2 (PGE2), produced by cyclooxygenase 2 (COX2) conversion of arachidonic acid, is one of the most important biologically active lipid mediators found throughout the gastrointestinal tract [117] and kidneys [118]. Works conducted on several animal models showed that PGE2 in the gastrointestinal (GI) tract acts through multiple prostaglandin E2 (EP) receptors and mediates important physiological processes such as gastric acid secretion, GI motility, and mucus secretion [117]. Additionally, increased levels of PGE2 detected in the GI mucosal membranes of patients and in experimental models of inflammatory bowel disease have been associated with improved epithelial repair [118]. In kidneys, PGE2 can exert protective or harmful effects through different target receptors [119]. Moreover, PGE2 is also a main regulator of liver function. It induces lipid accumulation in hepatocytes [120] and improves hepatic bile acid synthesis [121]. On the other hand, PGE2 exerts hepatoprotective effects against many liver injuries mainly by promoting proliferation or mitigating hepatocyte injury [122].

PGE2 is an important regulator of Na^+^/K^+^ ATPase activity in numerous cell types, including intestinal, kidney, and liver cells. However, limited evidence is available on the effect of PGE2 on the Na^+^/K^+^ ATPase trafficking.

### PGE2 and NKA trafficking in intestine

Nepal et al. [123] showed that in the small intestine, PGE2 reduces the Na^+^/K^+^ ATPase activity, In addition, the authors demonstrated by Western blot and immunofluorescent imaging of fixed cells that this inhibition is due to transcriptional repression and consequently to a decrease in the alpha1 and beta1-subunits at the plasma membrane. However, in this study, the decrease at the plasma membrane was associated with a decrease in the overall signal of Na^+^/K^+^ ATPase, suggesting that PGE2 causes a reduction in total Na^+^/K^+^ ATPase expression. Similarly, our group showed in Caco-2 cells that the pump’s activity was reduced by PGE2 and that this reduction was associated with lower protein levels of the Na^+^/K^+^ ATPase in both the cell homogenate and the membrane fraction [124], suggesting reduced localization of the pump at the plasma membrane. However, one interpretation of the results obtained could be that PGE2 regulates directly the trafficking and the translocation of the Na^+^/K^+^ ATPase to intracellular compartments where it is degraded; another interpretation could be that PGE2 reduces the overall expression of the pump. Consistent with these results, we demonstrated in a separate study using Caco-2 cells that FTY720P, an analogue of sphingosine 1-phosphate, mediates the inhibition of Na^+^/K^+^ ATPase activity by activating S1PR2 and inducing PGE2 release [125]. Recently, we showed, using confocal imaging of live Caco-2 cells co-expressing GFP-tagged alpha Na^+^/K^+^ ATPase and mcherry-tagged membrane marker, that FTY720P-mediated inhibition of the NKA pump results from a decrease in its abundance in the plasma membrane [13]. In addition to PGE2, FTY720P was found to induce NO release in these cells. Previous studies have shown that the vesicle fusing ATPase, N-ethylmaleimide sensitive factor (NSF) is a target for NO. It is also a major component of the trafficking machinery regulating vesicular targeting which includes, in addition to NSF, soluble NSF attachment receptor proteins (SNAREs), the Sec/Munc family, and the Rab family members [126]. Nitric oxide can also decelerate exocytosis by destabilizing the SNARE complex [127] and regulating small GTPases such as Ras and Rac that may lie upstream of the exocytic machinery [128]. Finally, by nitrosylation of ryanodine receptors on the sarcoplasmic reticulum (SR), NO can modulate cytosolic calcium levels that are important regulators of exocytosis [129, 130]. On the other hand, NO is known to accelerate endocytosis by enhancing S-nitrosylation of the GTPase dynamin which is well known to play an important role in the scission of nascent vesicles from the plasma membrane [131]. Whether FTY720P-mediated NO release is involved in the decrease in the amount of Na^+^/K^+^ ATPase at plasma membrane is still not clear.

### PGE2 and NKA trafficking in liver cells

Na^+^/K^+^ ATPase trafficking is also implicated in PGE2 regulation of the pump’s activity in liver cells. We previously showed that PGE2 enhances Na^+^/K^+^ ATPase activity in HepG2 through activation of EP4, production of cAMP, and PKA activation [12]. Our results also reported an important role for calcium released from intracellular stores in the PGE2 effect. Using pharmacological tools and fluorescent imaging, we uncovered the pivotal role of NKA trafficking in the PGE2-induced increase in its activity. Immunoblotting showed, in PGE2-treated cells, a higher expression of Na^+^/K^+^ ATPase alpha1 in cell membrane fractions but not in the whole cell homogenate, inferring that the increased abundance at the plasma membrane (PM) is not due to the increased protein expression. Interestingly, the stimulatory effect of PGE2 was completely blocked when cells were incubated on ice, demonstrating the dynamic nature of Na^+^/K^+^ ATPase vesicular transport. The PGE2 enhanced abundance of the pump at PM was also confirmed by confocal imaging of HepG2 cells expressing GFP-tagged Na^+^/K^+^ ATPase alpha1. Our time-lapse imaging also revealed a PGE2-induced highly dynamic pattern of Na^+^/K^+^ ATPase vesicles and a higher abundance of the pump at the cell membrane.

### PGE2 and NKA trafficking in the kidney

In the kidney, several reports demonstrated modulation of NKA activity by prostaglandins. Although PGE2 is the major prostaglandin synthesized in the kidney [132]. Herman et al. [133] reported in primary cultures of rabbit renal proximal tubule (RPT) cells, stimulation of the pump not only by PGE2 but also by other prostaglandins like PGE1 and PGF2α. The increased activity was reported in both short-term and long-term prostaglandin treatments. Long exposure of RPT cells to PGE1 and PGE2 resulted in an increase in Na^+^/K^+^ ATPase mRNA and total protein levels. Interestingly, under short-term treatments with PGE2, biotinylation assays of cells cultured on transwell membranes showed an increase in the level of NKA in the basolateral membrane without an increase in the overall level of cellular NKA. These results implicate a role for directional membrane trafficking in the regulation of NKA activity in these cells.

Similar findings were reported in Madin Darby Canine Kidney (MDCK) cells, where long exposures to PGE (4 h), acting via EP1 and EP2, induced an increase in mRNA and protein level of Na^+^/K^+^ ATPase [134, 135]. Although these studies demonstrated a role for Ca2 + in the PGE2-induced stimulation of the pump, the impact of calcium on pump trafficking was not studied. Interestingly, an independent work studying the short-term effect of PGE2 in MDCK showed an inhibition of the Na^+^/K^+^ ATPase, presumably due to impoverished residence at the plasma membrane rather than to changes in Na^+^/K^+^ ATPase expression [136].

Collectively, prior and current studies suggest that PGE2-modulation of the pump’s activity in liver, intestine, and kidney cells may be due to an effect on its trafficking and gene expression. However, more studies are required to address the molecular mechanisms linking PGE2-dependent modulation to Na^+^/K^+^ ATPase trafficking.

## Conclusions

The Na^+^/K^+^ ATPase is a vital enzyme in all types of animal cells. It maintains an essential ion gradient across the plasma membrane needed to drive normal cellular processes and sustain homeostasis. Alterations in the pump’s activity have been associated with several disorders including cardiovascular, neurological, kidney, and metabolic diseases [137]. Developing therapeutic strategies against these complications requires proper understanding of the processes involved in modulating directly or indirectly the activity of the pump, at both the cellular and molecular levels. Although the NKA pump has been extensively studied, the association between signal transduction pathways and its membrane trafficking has still not been fully elucidated. We tried in this review to focus on reported studies investigating the role of NKA trafficking in the modulation of the pump’s activity with an emphasis on the involvement of potential short-sequence endocytic motifs and phosphorylation sites.

## Data Availability

Not applicable.

## Abbreviations

R551: Arginine at position 551
D376: Aspartic acid at position D376
N158: Asparagine at position 158
N193: Asparagine at position 193
N265: Asparagine at position 265
[DE]xxxL[LI]: (D = aspartic acid, E = glutamic acid, x = any aminoacid, L = leucine, I = isoleucine)
[DE]-x(3)-Y: (D = aspartic acid, E = glutamic acid, x = any aminoacid, Y = tyrosine)
CCT: Chaperonin-containing TCP-1
FTY720P: Fingolimod (analogue sphingosine-1-phosphate)
E223: Glutamic acid at position 223
GI: Gastrointestinal
Caco-2: Human colorectal adenocarcinoma cells
HepG2: Human liver carcinoma cell
LL: Dileucine
MDCK: Madin darby canine kidney cells
NO: Nitric oxide
FXYD: Phenylalanine-any aminoacid-tyrosine-aspartic acid
PGE2: Prostaglandin E2
EP: Prostaglandin E2 receptor
PKC: Protein kinase C
NKA: Sodium–potassium pump or Na^+^/K^+^ ATPase
M2: Transmembrane domain 2
M3: Transmembrane domain 3
YxxL: (Y = tyrosine, x = any amoniacid, L = leucine)
YXXФ: (Y = tyrosine, x = any amoniacid, Ф = bulky hydrophobic residue)

## References

[1] Mobasheri A, Avila J, Cózar-Castellano I, Brownleader MD, Trevan M, Francis MJ, Lamb JF, Martín-Vasallo P (2000). Na+, K+-ATPase isozyme diversity; comparative biochemistry and physiological implications of novel functional interactions. Biosci Rep.

[2] Xie Z (2003). Molecular mechanisms of Na/K-ATPase-mediated signal transduction. Ann N Y Acad Sci.

[3] Scheiner-Bobis G (2002). The sodium pump. Its molecular properties and mechanics of ion transport. Eur J Biochem.

[4] Lingrel JB, Kuntzweiler T (1994). Na+, K(+)-ATPase. J Biol Chem.

[5] Suhail M (2010). Na, K-ATPase: ubiquitous multifunctional transmembrane protein and its relevance to various pathophysiological conditions. J Clin Med Res.

[6] Clausen MJ, Poulsen H (2013). Sodium/potassium homeostasis in the cell. Met Ions Life Sci.

[7] Clausen MV, Hilbers F, Poulsen H (2017). The structure and function of the Na, K- ATPase isoforms in health and disease. Front Physiol.

[8] Al-Khalili L, Yu M, Chibalin AV (2003). Na+, K+-ATPase trafficking in skeletal muscle: insulin stimulates translocation of both alpha 1- and alpha 2-subunit isoforms. FEBS Lett.

[9] Liu J, Shapiro JI (2007). Regulation of sodium pump endocytosis by cardiotonic steroids: molecular mechanisms and physiological implications. Pathophysiology.

[10] Lecuona E, Sun H, Vohwinkel C, Ciechanover A, Sznajder JI (2009). Ubiquitination participates in the lysosomal degradation of Na, K-ATPase in steady-state conditions. Am J Respir Cell Mol Biol.

[11] Alves DS, Thulin G, Loffing J, Kashgarian M, Caplan MJ (2015). Akt substrate of 160 kD regulates Na+, K+-ATPase trafficking in response to energy depletion and renal ischemia. J Am Soc Nephrol.

[12] Hodeify R, Chakkour M, Rida R, Kreydiyyeh S (2021). PGE2 upregulates the Na+/K+ ATPase in HepG2 cells via EP4 receptors and intracellular calcium. PLoS ONE.

[13] Rida R, Hodeify R, Kreydiyyeh S (2023). Adverse effect of FTY720P on colonic Na+ /K+ ATPase is mediated via ERK, p38MAPK, PKC, and PI3K. J Appl Toxicol.

[14] Chibalin AV, Katz AI, Berggren PO, Bertorello AM (1997). Receptor-mediated inhibition of renal Na(+)-K(+)-ATPase is associated with endocytosis of its alpha- and beta-subunits. Am J Physiol.

[15] Zhang Y, Norian JM, Magyar CE, Holstein-Rathlou NH, Mircheff AK, McDonough AA (1999). In vivo PTH provokes apical NHE3 and NaPi2 redistribution and Na-K- ATPase inhibition. Am J Physiol.

[16] Feldmann T, Glukmann V, Medvenev E, Shpolansky U, Galili D, Lichtstein D, Rosen H (2007). Role of endosomal Na+-K+-ATPase and cardiac steroids in the regulation of endocytosis. Am J Physiol Cell Physiol.

[17] Cherniavsky-Lev M, Golani O, Karlish SJ, Garty H (2014). Ouabain-induced internalization and lysosomal degradation of the Na+/K+-ATPase. J Biol Chem.

[18] Khajah MA, Mathew PM, Luqmani YA (2018). Na+/K+ ATPase activity promotes invasion of endocrine resistant breast cancer cells. PLoS ONE.

[19] Leu WJ, Wang CT, Hsu JL, Chen IS, Chang HS, Guh JH (2020). Ascleposide, a natural cardenolide, induces anticancer signaling in human castration-resistant prostatic cancer through Na+ /K+ -ATPase internalization and tubulin acetylation. Prostate.

[20] Dada LA, Chandel NS, Ridge KM, Pedemonte C, Bertorello AM, Sznajder JI (2003). Hypoxia-induced endocytosis of Na, K-ATPase in alveolar epithelial cells is mediated by mitochondrial reactive oxygen species and PKC-zeta. J Clin Invest.

[21] Welch LC, Lecuona E, Briva A, Trejo HE, Dada LA, Sznajder JI (2010). Extracellular signal-regulated kinase (ERK) participates in the hypercapnia-induced Na K-ATPase downregulation. FEBS Lett.

[22] Berger G, Guetta J, Klorin G, Badarneh R, Braun E, Brod V (2011). Sepsis impairs alveolar epithelial function by downregulating Na-K-ATPase pump. Am J Physiol Lung Cell Mol Physiol.

[23] Bhatia T, Cornelius F, Brewer J, Bagatolli LA, Simonsen AC, Ipsen JH, Mouritsen OG (2016). Spatial distribution and activity of Na(+)/K(+)-ATPase in lipid bilayer membranes with phase boundaries. Biochim Biophys Acta.

[24] Omasits U, Ahrens CH, Müller S, Wollscheid B (2014). Protter: interactive protein feature visualization and integration with experimental proteomic data. Bioinformatics.

[25] Morth JP, Pedersen BP, Toustrup-Jensen MS, Sørensen TL, Petersen J, Andersen JP (2007). Crystal structure of the sodium-potassium pump. Nature.

[26] Kaplan JH (2002). Biochemistry of Na, K-ATPase. Annu Rev Biochem.

[27] Antolovic R, Brüller HJ, Bunk S, Linder D, Schoner W (1991). Epitope mapping by amino-acid-sequence-specific antibodies reveals that both ends of the alpha subunit of Na+/K(+)-ATPase are located on the cytoplasmic side of the membrane. Eur J Biochem.

[28] Jorgensen PL, Hakansson KO, Karlish SJ (2003). Structure and mechanism of Na, K- ATPase: functional sites and their interactions. Annu Rev Physiol.

[29] Shinoda T, Ogawa H, Cornelius F, Toyoshima C (2009). Crystal structure of the sodium-potassium pump at 2.4 a resolution. Nature.

[30] Therien AG, Nestor NB, Ball WJ, Blostein R (1996). Tissue-specific versus isoform- specific differences in cation activation kinetics of the Na K-ATPase. J Biol Chem.

[31] Blanco G, Mercer RW (1998). Isozymes of the Na-K-ATPase: heterogeneity in structure, diversity in function. Am J Physiol.

[32] Miller RP, Farley RA (1988). All three potential N-glycosylation sites of the dog kidney (Na+ + K+)-ATPase beta-subunit contain oligosaccharide. Biochim Biophys Acta.

[33] Kryvenko V, Vagin O, Dada LA, Sznajder JI, Vadász I (2021). Maturation of the Na, K-ATPase in the endoplasmic reticulum in health and disease. J Membr Biol.

[34] Lemas MV, Yu HY, Takeyasu K, Kone B, Fambrough DM (1994). Assembly of Na, K-ATPase alpha-subunit isoforms with Na, K-ATPase beta-subunit isoforms and H K- ATPase beta-subunit. J Biol Chem.

[35] Xie Z, Wang Y, Liu G, Zolotarjova N, Periyasamy SM, Askari A (1996). Similarities and differences between the properties of native and recombinant Na+/K+-ATPases. Arch Biochem Biophys.

[36] Gatto C, McLoud SM, Kaplan JH (2001). Heterologous expression of Na(+)-K(+)- ATPase in insect cells: intracellular distribution of pump subunits. Am J Physiol Cell Physiol.

[37] Geering K, Meyer DI, Paccolat MP, Kraehenbühl JP, Rossier BC (1985). Membrane insertion of alpha- and beta-subunits of Na+, K+-ATPase. J Biol Chem.

[38] Ackermann U, Geering K (1990). Mutual dependence of Na, K-ATPase alpha- and beta-subunits for correct posttranslational processing and intracellular transport. FEBS Lett.

[39] Durlacher CT, Chow K, Chen XW, He ZX, Zhang X, Yang T, Zhou SF (2015). Targeting Na^+^/K^+^ -translocating adenosine triphosphatase in cancer treatment. Clin Exp Pharmacol Physiol.

[40] Geering K (1991). The functional role of the beta-subunit in the maturation and intracellular transport of Na K-ATPase. FEBS Lett.

[41] Lutsenko S, Kaplan JH (1993). An essential role for the extracellular domain of the Na K-ATPase beta-subunit in cation occlusion. Biochemistry.

[42] Forbush B, Kaplan JH, Hoffman JF (1978). Characterization of a new photoaffinity derivative of ouabain: labeling of the large polypeptide and of a proteolipid component of the Na K-ATPase. Biochemistry.

[43] Therien AG, Blostein R (2000). Mechanisms of sodium pump regulation. Am J Physiol Cell Physiol.

[44] Geering K (2006). FXYD proteins: new regulators of Na-K-ATPase. Am J Physiol Renal Physiol.

[45] Arystarkhova E, Wetzel RK, Asinovski NK, Sweadner KJ (1999). The gamma subunit modulates Na(+) and K(+) affinity of the renal Na. K-ATPase J Biol Chem.

[46] Houy S, Croisé P, Gubar O, Chasserot-Golaz S, Tryoen-Tóth P, Bailly Y, Ory S, Bader MF, Gasman S (2013). Exocytosis and endocytosis in neuroendocrine cells: inseparable membranes!. Front Endocrinol.

[47] Cai H, Reinisch K, Ferro-Novick S (2007). Coats, tethers, Rabs, and SNAREs work together to mediate the intracellular destination of a transport vesicle. Dev Cell.

[48] Cui L, Li H, Xi Y, Hu Q, Liu H, Fan J, Xiang Y, Zhang X, Shui W, Lai Y (2022). Vesicle trafficking and vesicle fusion: mechanisms, biological functions, and their implications for potential disease therapy. Molecular biomedicine.

[49] Mancias JD, Goldberg J (2008). Structural basis of cargo membrane protein discrimination by the human COPII coat machinery. EMBO J.

[50] Zanetti G, Prinz S, Daum S, Meister A, Schekman R, Bacia K, Briggs JA (2013). The structure of the COPII transport-vesicle coat assembled on membranes. Elife.

[51] Miller EA, Beilharz TH, Malkus PN, Lee MC, Hamamoto S, Orci L, Schekman R (2003). Multiple cargo binding sites on the COPII subunit Sec24p ensure capture of diverse membrane proteins into transport vesicles. Cell.

[52] De Matteis MA, Luini A (2008). Exiting the Golgi complex. Nat Rev Mol Cell Biol.

[53] Mattera R, Boehm M, Chaudhuri R, Prabhu Y, Bonifacino JS (2011). Conservation and diversifcation of dileucine signal recognition by adaptor protein (AP) complex variants. J Biol Chem.

[54] Janvier K, Kato Y, Boehm M, Rose JR, Martina JA, Kim BY (2003). Recognition of dileucine-based sorting signals from HIV-1 Nef and LIMP-II by the AP-1 γ–σ1 and AP-3 δ–σ3 hemicomplexes. J Cell Biol.

[55] McMahon HT, Boucrot E (2011). Molecular mechanism and physiological functions of clathrin-mediated endocytosis. Nat Rev Mol Cell Biol.

[56] Schmid SL, Frolov VA (2011). Dynamin: functional design of a membrane fission catalyst. Annu Rev Cell Dev Biol.

[57] Nabi IR, Le PU (2003). Caveolae/raft-dependent endocytosis. J Cell Biol.

[58] Matthaeus C, Taraska JW (2021). Energy and Dynamics of Caveolae Trafficking. Front Cell Dev Biol.

[59] Collins BM, Davis MJ, Hancock JF, Parton RG (2012). Structure-based reassessment of the caveolin signaling model: do caveolae regulate signaling through caveolin-protein interactions?. Dev Cell.

[60] Devarajan P, Stabach PR, De Matteis MA, Morrow JS (1997). Na, K-ATPase transport from endoplasmic reticulum to Golgi requires the Golgi spectrin-ankyrin G119 skeleton in Madin Darby canine kidney cells. Proc Natl Acad Sci USA.

[61] Beggah A, Mathews P, Beguin P, Geering K (1996). Degradation and endoplasmic reticulum retention of unassembled alpha- and beta-subunits of Na, K- ATPase correlate with interaction of BiP. J Biol Chem.

[62] Beggah AT, Geering K (1997). Alpha and beta subunits of Na, K-ATPase interact with BiP and calnexin. Ann N Y Acad Sci.

[63] Tokhtaeva E, Sachs G, Vagin O (2010). Diverse pathways for maturation of the Na, K-ATPase β1 and β2 subunits in the endoplasmic reticulum of Madin-Darby canine kidney cells. J Biol Chem.

[64] Tokhtaeva E, Munson K, Sachs G, Vagin O (2010). N-glycan-dependent quality control of the Na, K-ATPase beta(2) subunit. Biochemistry.

[65] Laughery MD, Todd ML, Kaplan JH (2003). Mutational analysis of alpha- beta subunit interactions in the delivery of Na, K-ATPase heterodimers to the plasma membrane. J Biol Chem.

[66] Sznajder JI, Factor P, Ingbar DH (2002). Lung edema clearance: role of Na+-K+-ATPase. J Appl Physiol.

[67] Bertorello AM, Ridge KM, Chibalin AV, Katz AI, Sznajder JI (1999). Isoproterenol increases Na+-K+-ATPase activity by membrane insertion of alpha- subunits in lung alveolar cells. Am J Physiol.

[68] Ridge KM, Dada L, Lecuona E, Bertorello AM, Katz AI, Mochly-Rosen D, Sznajder JI (2002). Dopamine-induced exocytosis of Na, K-ATPase is dependent on activation of protein kinase C-epsilon and -delta. Mol Biol Cell.

[69] Hundal HS, Marette A, Mitsumoto Y, Ramlal T, Blostein R, Klip A (1992). Insulin induces translocation of the alpha 2 and beta 1 subunits of the Na+/K(+)- ATPase from intracellular compartments to the plasma membrane in mammalian skeletal muscle. J Biol Chem.

[70] Sweeney G, Niu W, Canfield VA, Levenson R, Klip A (2001). Insulin increases plasma membrane content and reduces phosphorylation of Na(+)-K(+) pump alpha(1)-subunit in HEK-293 cells. Am J Physiol Cell Physiol.

[71] Wang X, Cai L, Xie JX, Cui X, Zhang J, Wang J, Chen Y, Larre I, Shapiro JI, Pierre SV, Wu D, Zhu GZ, Xie Z (2020). A caveolin binding motif in Na/K-ATPase is required for stem cell differentiation and organogenesis in mammals and Celegans. Sci Adv.

[72] Elkin SR, Lakoduk AM, Schmid SL (2016). Endocytic pathways and endosomal trafficking: a primer. Wien Med Wochenschr.

[73] Otto GP, Nichols BJ (2011). The roles of flotillin microdomains–endocytosis and beyond. J Cell Sci.

[74] Glebov OO, Bright NA, Nichols BJ (2006). Flotillin-1 defines a clathrin- independent endocytic pathway in mammalian cells. Nat Cell Biol.

[75] John BA, Meister M, Banning A, Tikkanen R (2014). Flotillins bind to the dileucine sorting motif of β-site amyloid precursor protein-cleaving enzyme 1 and influence its endosomal sorting. FEBS J.

[76] Chibalin AV, Pedemonte CH, Katz AI, Féraille E, Berggren PO, Bertorello AM (1998). Phosphorylation of the catalyic alpha-subunit constitutes a triggering signal for Na+, K+- ATPase endocytosis. J Biol Chem.

[77] Liu J, Kesiry R, Periyasamy SM, Malhotra D, Xie Z, Shapiro JI (2004). Ouabain induces endocytosis of plasmalemmal Na/K-ATPase in LLC-PK1 cells by a clathrin- dependent mechanism. Kidney Int.

[78] Chibalin AV, Ogimoto G, Pedemonte CH, Pressley TA, Katz AI, Féraille E, Berggren PO, Bertorello AM (1999). Dopamine-induced endocytosis of Na+, K+-ATPase is initiated by phosphorylation of Ser-18 in the rat alpha subunit and Is responsible for the decreased activity in epithelial cells. J Biol Chem.

[79] Sottejeau Y, Belliard A, Duran MJ, Pressley TA, Pierre SV (2010). Critical role of the isoform-specific region in alpha1-Na, K-ATPase trafficking and protein Kinase C- dependent regulation. Biochemistry.

[80] Efendiev R, Budu CE, Bertorello AM, Pedemonte CH (2008). G-protein- coupled receptor-mediated traffic of Na, K-ATPase to the plasma membrane requires the binding of adaptor protein 1 to a Tyr-255-based sequence in the alpha-subunit. J Biol Chem.

[81] Pierre SV, Belliard A, Sottejeau Y (2011). Modulation of Na(+)-K(+)-ATPase cell surface abundance through structural determinants on the α1-subunit. Am J Physiol Cell Physiol.

[82] Alzamora R, Marusic ET, Gonzalez M, Michea L (2003). Nongenomic effect of aldosterone on Na+, K+-adenosine triphosphatase in arterial vessels. Endocrinology.

[83] Yin J, Guo HC, Yu D, Wang HC, Li JX, Wang YL (2014). Mechanisms of isoform-specific Na/K pump regulation by short- and long-term adrenergic activation in rat ventricular myocytes. Cell Physiol Biochem.

[84] Zhang J, Li X, Yu H, Larre I, Dube PR, Kennedy DJ, Tang WHW, Westfall K, Pierre SV, Xie Z, Chen Y (2020). Regulation of Na/K-ATPase expression by cholesterol: isoform specificity and the molecular mechanism. Am J Physiol Cell Physiol.

[85] Pandey KN (2009). Functional roles of short sequence motifs in the endocytosis of membrane receptors. Front Biosci (Landmark Ed).

[86] Mousavi SA, Malerød L, Berg T, Kjeken R (2004). Clathrin-dependent endocytosis. Biochem J.

[87] Doné SC, Leibiger IB, Efendiev R, Katz AI, Leibiger B, Berggren PO, Pedemonte CH, Bertorello AM (2002). Tyrosine 537 within the Na+, K+-ATPase alpha-subunit is essential for AP-2 binding and clathrin-dependent endocytosis. J Biol Chem.

[88] Bonifacino JS, Traub LM (2003). Signals for sorting of transmembrane proteins to endosomes and lysosomes. Annu Rev Biochem.

[89] Ray A, Katoch P, Jain N, Mehta PP (2018). Dileucine-like motifs in the C-terminal tail of connexin32 control its endocytosis and assembly into gap junctions. J Cell Sci.

[90] Kimura T, Allen PB, Nairn AC, Caplan MJ (2007). Arrestins and spinophilin competitively regulate Na+, K+-ATPase trafficking through association with a large cytoplasmic loop of the Na+, K+-ATPase. Mol Biol Cell.

[91] Stephens DJ, Banting G (1999). Direct interaction of the trans-Golgi network membrane protein, TGN38, with the F-actin binding protein, neurabin. J Biol Chem.

[92] Lecuona E, Dada LA, Sun H, Butti ML, Zhou G, Chew TL, Sznajder JI (2006). Na, K- ATPase alpha1-subunit dephosphorylation by protein phosphatase 2A is necessary for its recruitment to the plasma membrane. FASEB J.

[93] Liu J, Liang M, Liu L, Malhotra D, Xie Z, Shapiro JI (2005). Ouabain-induced endocytosis of the plasmalemmal Na/K-ATPase in LLC-PK1 cells requires caveolin-1. Kidney Int.

[94] Shigematsu S, Watson RT, Khan AH, Pessin JE (2003). The adipocyte plasma membrane caveolin functional/structural organization is necessary for the efficient endocytosis of GLUT4. J Biol Chem.

[95] Sleight S, Wilson BA, Heimark DB, Larner J (2002). G(q/11) is involved in insulin- stimulated inositol phosphoglycan putative mediator generation in rat liver membranes: co-localization of G(q/11) with the insulin receptor in membrane vesicles. Biochem Biophys Res Commun.

[96] Scherer PE, Lisanti MP, Baldini G, Sargiacomo M, Mastick CC, Lodish HF (1994). Induction of caveolin during adipogenesis and association of GLUT4 with caveolin-rich vesicles. J Cell Biol.

[97] Stoddart A, Dykstra ML, Brown BK, Song W, Pierce SK, Brodsky FM (2002). Lipid rafts unite signaling cascades with clathrin to regulate BCR internalization. Immunity.

[98] Larance M, Ramm G, Stöckli J, van Dam EM, Winata S, Wasinger V (2005). Characterization of the role of the Rab GTPase-activating protein AS160 in insulin- regulated GLUT4 trafficking. J Biol Chem.

[99] Alves DS, Farr GA, Seo-Mayer P, Caplan MJ (2010). AS160 associates with the Na+, K+- ATPase and mediates the adenosine monophosphate-stimulated protein kinase-dependent regulation of sodium pump surface expression. Mol Biol Cell.

[100] Morton MJ, Farr GA, Hull M, Capendeguy O, Horisberger JD, Caplan MJ (2010). Association with {beta}-COP regulates the trafficking of the newly synthesized Na. K- ATPase J Biol Chem.

[101] Kozik P, Francis RW, Seaman MN, Robinson MS (2010). A screen for endocytic motifs. Traffic.

[102] Marks MS, Ohno H, Kirchnausen T, Bonracino JS (1997). Protein sorting by tyrosine- based signals: adapting to the Ys and wherefores. Trends Cell Biol.

[103] Hodeify R, Nandakumar M, Own M, Courjaret RJ, Graumann J, Hubrack SZ, Machaca K (2018). The CCT chaperonin is a novel regulator of Ca2+ signaling through modulation of Orai1 trafficking. Sci Adv.

[104] Kubota H, Hynes G, Willison K (1995). The chaperonin containing t-complex polypeptide 1 (TCP-1). Multisubunit machinery assisting in protein folding and assembly in the eukaryotic cytosol. Eur J Biochem.

[105] Bakthavatsalam D, Soung RH, Tweardy DJ, Chiu W, Dixon RA, Woodside DG (2014). Chaperonin-containing TCP-1 complex directly binds to the cytoplasmic domain of the LOX-1 receptor. FEBS Lett.

[106] Poulsen H, Morth P, Egebjerg J, Nissen P (2010). Phosphorylation of the Na+, K+- ATPase and the H+, K+-ATPase. FEBS Lett.

[107] Bibert S, Roy S, Schaer D, Horisberger JD, Geering K (2008). Phosphorylation of phospholemman (FXYD1) by protein kinases A and C modulates distinct Na. K-ATPase isozymes J Biol Chem.

[108] Teriete P, Thai K, Choi J, Marassi FM (2009). Effects of PKA phosphorylation on the conformation of the Na, K-ATPase regulatory protein FXYD1. Biochim Biophys Acta.

[109] Feschenko MS, Sweadner KJ (1997). Phosphorylation of Na, K-ATPase by protein kinase C at Ser18 occurs in intact cells but does not result in direct inhibition of ATP hydrolysis. J Biol Chem.

[110] Kava L, Rossi NF, Mattingly R, Yingst DR (2012). Increased expression of Na, K- ATPase and a selective increase in phosphorylation at Ser-11 in the cortex of the 2- kidney, 1-clip hypertensive rat. Am J Hypertens.

[111] Einholm AP, Nielsen HN, Holm R, Toustrup-Jensen MS, Vilsen B (2016). Importance of a Potential Protein Kinase A Phosphorylation Site of Na+, K+-ATPase and Its Interaction Network for Na+ Binding. J Biol Chem.

[112] Massey KJ, Li Q, Rossi NF, Keezer SM, Mattingly RR, Yingst DR (2016). Phosphorylation of rat kidney Na-K pump at Ser938 is required for rapid angiotensin II- dependent stimulation of activity and trafficking in proximal tubule cells. Am J Physiol Cell Physiol.

[113] Chen Z, Krmar RT, Dada L, Efendiev R, Leibiger IB, Pedemonte CH, Katz AI, Sznajder JI, Bertorello AM (2006). Phosphorylation of adaptor protein-2 mu2 is essential for Na+, K+-ATPase endocytosis in response to either G protein-coupled receptor or reactive oxygen species. Am J Respir Cell Mol Biol.

[114] Rust HL, Thompson PR (2011). Kinase consensus sequences: a breeding ground for crosstalk. ACS Chem Biol.

[115] Patschinsky T, Hunter T, Esch FS, Cooper JA, Sefton BM (1982). Analysis of the sequence of amino acids surrounding sites of tyrosine phosphorylation. Proc Natl Acad Sci U S A.

[116] Billet A, Jia Y, Jensen TJ, Hou YX, Chang XB, Riordan JR, Hanrahan JW (2016). Potential sites of CFTR activation by tyrosine kinases. Channels (Austin).

[117] Dey I, Lejeune M, Chadee K (2006). Prostaglandin E2 receptor distribution and function in the gastrointestinal tract. Br J Pharmacol.

[118] Mutsaers HAM, Nørregaard R (2022). Prostaglandin E2 receptors as therapeutic targets in renal fibrosis. Kidney Res Clin Pract.

[119] Yang C, Li C, Li M, Tong X, Hu X, Yang X, Yan X, He L, Wan C (2015). CYP2S1 depletion enhances colorectal cell proliferation is associated with PGE2-mediated activation of β-catenin signaling. Exp Cell Res.

[120] Nasrallah R, Hassouneh R, Hébert RL (2016). PGE2, Kidney Disease, and Cardiovascular Risk: Beyond Hypertension and Diabetes. J Am Soc Nephrol.

[121] Henkel J, Frede K, Schanze N, Vogel H, Schürmann A, Spruss A, Bergheim I, Püschel GP (2012). Stimulation of fat accumulation in hepatocytes by PGE_2_-dependent repression of hepatic lipolysis, β-oxidation and VLDL-synthesis. Lab Invest.

[122] Yan S, Tang J, Zhang Y, Wang Y, Zuo S, Shen Y (2017). Prostaglandin E2 promotes hepatic bile acid synthesis by an E prostanoid receptor 3-mediated hepatocyte nuclear receptor 4α/cholesterol 7α-hydroxylase pathway in mice. Hepatology.

[123] Nepal N, Arthur S, Haynes J, Palaniappan B, Sundaram U (2021). Mechanism of Na- K-ATPase Inhibition by PGE2 in Intestinal Epithelial Cells. Cells.

[124] Elmoussawi L, Chakkour M, Kreydiyyeh S (2019). The epinephrine-induced PGE2 reduces Na+/K+ ATPase activity in Caco-2 cells via PKC NF-κB and NO. PLoS ONE.

[125] Rida R, Kreydiyyeh S (2018). FTY720P inhibits the Na+/K+ ATPase in Caco-2 cells via S1PR2: PGE2 and NO are along the signaling pathway. Life Sci.

[126] Lowenstein CJ (2007). Nitric oxide regulation of protein trafficking in the cardiovascular system. Cardiovasc Res.

[127] Meffert MK, Calakos NC, Scheller RH, Schulman H (1996). Nitric oxide modulates synaptic vesicle docking fusion reactions. Neuron.

[128] Ferlito M, Irani K, Faraday N, Lowenstein CJ (2006). Nitric oxide inhibits exocytosis of cytolytic granules from lymphokine-activated killer cells. Proc Natl Acad Sci U S A.

[129] Xu L, Eu JP, Meissner G, Stamler JS (1998). Activation of the cardiac calcium release channel (ryanodine receptor) by poly-S-nitrosylation. Science.

[130] Gonzalez DR, Beigi F, Treuer AV, Hare JM (2007). Deficient ryanodine receptor S- nitrosylation increases sarcoplasmic reticulum calcium leak and arrhythmogenesis in cardiomyocytes. Proc Natl Acad Sci U S A.

[131] Kang-Decker N, Cao S, Chatterjee S, Yao J, Egan LJ, Semela D, Mukhopadhyay D, Shah V (2007). Nitric oxide promotes endothelial cell survival signaling through S- nitrosylation and activation of dynamin-2. J Cell Sci.

[132] Bonvalet JP, Pradelles P, Farman N (1987). Segmental synthesis and actions of prostaglandins along the nephron. Am J Physiol.

[133] Herman MB, Rajkhowa T, Cutuli F, Springate JE, Taub M (2010). Regulation of renal proximal tubule Na-K-ATPase by prostaglandins. Am J Physiol Renal Physiol.

[134] Matlhagela K, Taub M (2006). Involvement of EP1 and EP2 receptors in the regulation of the Na, K-ATPase by prostaglandins in MDCK cells. Prostaglandins Other Lipid Mediat.

[135] Taub M, Borsick M, Geisel J, Matlhagela K, Rajkhowa T, Allen C (2004). Regulation of the Na, K-ATPase in MDCK cells by prostaglandin E1: a role for calcium as well as cAMP. Exp Cell Res.

[136] Cohen-Luria R, Rimon G, Moran A (1993). PGE2 inhibits Na-K-ATPase activity and ouabain binding in MDCK cells. Am J Physiol.

[137] Yan Y, Shapiro JI (2016). The physiological and clinical importance of sodium potassium ATPase in cardiovascular diseases. Curr Opin Pharmacol.

